# Research on Spatial Unbalance and Influencing Factors of Ecological Well-Being Performance in China

**DOI:** 10.3390/ijerph18179299

**Published:** 2021-09-03

**Authors:** Shengyun Wang, Yaxin Zhang, Xingren Yao

**Affiliations:** 1 Research Center of the Central China for Economic and Social Development, Nanchang University, Nanchang 330031, China; wangshengyun@163.com; 2 School of Economics and Management, Nanchang University, Nanchang 330031, China; chinayao16@163.com

**Keywords:** ecological well-being performance, spatial difference, super-SBM, Dagum Gini coefficient decomposition, geographically and temporally weighted regression

## Abstract

Ecological well-being performance (EWP) is central to achieving coordinated and sustainable economic and social development and environmental protection. This study constructed an evaluation index system of EWP, measured the EWP of 30 Chinese provinces or cities from 1997 to 2018 using the super-efficiency slack-based model (Super-SBM), and analyzed the spatial and temporal evolutionary characteristics of EWP. Under the division of four regions and eight regions, the Dagum Gini coefficient decomposition is applied to analyze the regional differences and sources of differences in EWP in China. Then, the Geographically and Temporally Weighted Regression (GTWR) model analyzes the factors influencing EWP. Results show that the inter-provincial differences in EWP in China are significant, with the eastern region having significantly higher EWP than the western, central, and northeastern regions. From 1997 to 2018, the overall spatial differences in EWP in China decreased. The four regions and eight regions show that reducing inter-regional differences is the key to mitigating regional unbalance in China. Urbanization significantly enhances EWP in China and the degree of openness and industrial structure has a significant heterogeneous effect on EWP. Therefore, future policy formulation should focus on transforming the economic development model, promoting coordinated regional development, and exploring the optimal ways to improve EWP according to local conditions. This study aims to provide a scientific basis and reference for promoting sustainable regional economic and social development and improving the imbalance.

## 1. Introduction

The rapid growth of China’s economy is accompanied by intensified environmental pollution [1,2] and unbalanced regional development [3]. The economic growth model at the cost of environmental pollution enhances human economic well-being and reduces environmental well-being from sustainable development [4], harming overall human well-being. In the new era, China’s economy has entered the stage of high-quality development [5] and promoting people’s well-being has become the fundamental purpose of development. Thus, China must explore a high-quality development path that synergizes economic and social development with environmental protection, shifting its focus from improving people’s economic well-being to enhancing their overall well-being. Therefore, simultaneously building a well-being performance evaluation system that can characterize economic, social, and environmental (i.e., ecological well-being performance (EWP)) elements is crucial to improve people’s comprehensive well-being with the minimum consumption of resources. Most existing studies have been conducted to measure a country’s well-being level or region from economic well-being and social well-being, but many of them do not focus on the comprehensive well-being that includes economic, social, and environmental elements. How to coordinate the relationship among economic growth, social development, and environmental protection and seek a new way of high-quality development based on low consumption, low pollution, and high well-being has become a vital issue in China’s current economic and social development. The quantitative measurement of regional differences in EWP is an essential reference for promoting high-quality, coordinated regional development in China. Furthermore, based on measuring EWP, exploring the influencing factors of EWP can also provide possible suggestions for China to develop differentiated strategies to improve EWP.

Maximizing comprehensive well-being is an inevitable choice to achieve sustainable development [6], and the scarcity of natural capital has become an essential factor limiting human development [7]. The focus of attention is how to deal with the relationship among resource consumption, economic growth, and well-being improvement within the ecological boundary [8]. To achieve the win–win goal of coordinating economic and social development with environmental protection and human well-being improvement, assessing the level of EWP is crucial. The concept of EWP was first introduced by Daly (1974) [9] to assess the efficiency of natural consumption for enhancing well-being. EWP reflects the degree of harmony between the relative changes in human well-being improvement and ecological resource consumption. Assessing EWP for coordinating economic, social, and environmental development and well-being improvement has become the focus of government and economic and environmental researchers [10]. In addition, exploring the sources and influencing factors of regional differences in EWP is necessary to provide a reference for promoting coordinated regional development and improving EWP.

This study aims to construct a comprehensive and objective EWP evaluation index system and measure the EWP of 30 provinces in China by using the Super-SBM model. It helps grasp the current situation of EWP in China, deepen people’s understanding of EWP, and promote sustainable economic and social development. Moreover, an in-depth analysis of the spatial unbalance of EWP is conducted, and its sources of difference can clarify the policy focus points for promoting the formation of regional synergistic development patterns in the future. Finally, the driving factors of EWP are analyzed to explore the paths to enhance EWP. This study has theoretical and practical significance for the sustainable development of China’s economy and society. It provides a reference for other countries to quantitatively evaluate EWP and improve the unbalanced EWP.

## 2. Literature Review

**Figure 1:**
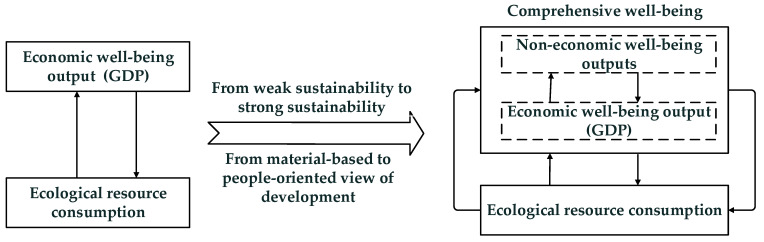
From eco-efficiency to ecological well-being performance analysis framework.

The economic system is a subsystem of the ecosystem [11], and the ecological boundary constrains the expansion of the economic system. Human beings have changed from an “empty world,” where artificial capital is relatively scarce, to a “full world,” where natural capital is scarce. In a “full world,” the constraint of resources and environment on economic growth is becoming increasingly apparent, and the development of society must be achieved within the ecological carrying capacity [12]. It belongs to the category of strong, sustainable development. The traditional pursuit of economic performance based on the consumption of ecological resources, that is, eco-efficiency, still belongs to weak sustainable development [13]. In contrast to weak sustainability, strong sustainability emphasizes the non-decreasing nature of key natural capital and argues that man-made capital cannot wholly replace natural capital [14]. Based on the theory of strong sustainability, social development must increase the comprehensive well-being level, including economic, social, and environmental well-being with the least possible ecological resource consumption under the ecological threshold constraint, that is, improving EWP. It also realizes the change from a material-based view of pursuing ecological efficiency to a human-centered view of development pursuing EWP. We established an analytical framework (Figure 1) for EWP based on the article by Zang et al. (2013) [13].

The concept of EWP was first proposed by Daly (1974), who defined the low entropy resources consumed, high entropy waste released to the environment as throughput, the utility or well-being obtained from the ecosystem as service, and the ratio of service to throughput to characterize the efficiency of resource consumption into well-being level [9]. However, Daly did not propose specific indicators to quantify service volume and throughput, making the concept of EWP not widely applied.

Well-being maximization is a socially accepted goal, and GDP is often used as a proxy for the relative efficiency of an economy. However, using the amount of GDP as a measure of a country or region has been highly controversial [15,16]. From the perspective of EWP, GDP also covers its economic dimension only. The Human Development Index (HDI) [17] proposed by the United Nations Development Program in 1990 has extended the well-being indicator from the economic dimension to the social dimension and enabled the quantification of well-being levels. However, all high-level countries have unsustainable ecological impacts, and no indicators cover ecological sustainability in HDI calculation [18]. In addition to HDI, happy life years [16], life expectancy at birth [19], and sustainable economic well-being index [20] are also used as indicators of well-being levels. They go beyond GDP to measure the utility that people derive from ecosystems. Regardless of the indicator used to measure the level of well-being, in addition to economic growth, social and environmental issues should be considered, including education, health, and environmental variables [21].

Rees (1992) introduced the concept of ecological footprint to quantify natural consumption in 1992 [22]. According to Wackernagel and Rees (1998) [23], the ecological footprint is calculated from six types of land area, and this method calculates the size of resources that humans obtain from ecosystems. The ecological footprint can measure the natural consumption of a country or region over a certain period. For example, Haberl et al. (2001) calculated and explored the ecological footprint of Austria in 1926–1995 [24], Mcdonald and Patterson (2004) calculated the ecological footprint of the Auckland region using input–output analysis [25], and Yin et al. (2017) calculated the ecological footprint of five provinces in northwest China in 2005–2014 and compared it with the regional development capacity [26]. The materials and energy provided by the ecosystem are the material basis of a region’s development. Economic and social development is achieved through labor and capital input while exporting waste to the environment [16]. Therefore, when evaluating the EWP of a country or region, elements such as labor and capital, which achieve resource utilization, must be considered. An ecological footprint is only a tool for evaluating the state of environmental stress in a region [27], and its calculation formula does not include the inputs of labor and capital. EWP is a concept covering economic, social, and environmental dimensions and is a complex system with multiple inputs and outputs. The input must cover resource consumption and labor and capital elements to achieve resource utilization [28].

Studies on EWP have emerged after quantifying “service volume” and “throughput” (Table 1). It is mainly manifested in the following three aspects. Firstly, ecological well-being performance (EWP) is measured and analyzed. Measuring EWP has two main approaches. The first is based on the ratio of social well-being to the ecological footprint or resource consumption. For example, Abdallah et al. (2009) defined the ratio of the happy life index to ecological footprint as the Happy Planet Index and used it to measure sustainable development in 143 countries [29]. Zhang et al. (2018) constructed an EWP model using the ratio of HDI to ecological footprint and measured the EWP of countries with a population of 10 million or more [30]. The second one is based on the Stochastic Frontier Approach (SFA) and Data Envelopment Analysis (DEA). Dietz et al. (2009) used SFA to measure the well-being performance of 135 countries [31]. Bian et al. (2020) measured the EWP of 30 provincial capitals in China by Super-SBM model and found that the EWP showed a spatial distribution pattern of the firm in the east and a weak one in the west [32]. Ibrahim et al. (2021) measured the socio-ecological efficiency of sub-Saharan African countries using the DEA approach [33]. Yao et al. (2021) assessed the EWP of 30 Chinese provinces through the Super-SBM model and analyzed the spatial correlation of eco-well-being performance through the Moran index [3]. Second, regional differences in EWP are investigated. Wang and Feng (2020) calculate the Theil index for three regions in China and find that interregional differences contribute the most to the overall differences [34]. Li et al. (2020) analyzed ecological total factor productivity in China and find spatial convergence [1]. Third, the influencing factors of EWP are explored. Zhang et al. (2020) apply a panel data model to investigate the influencing factors of ecological consumption [35].

Existing studies provide theoretical and methodological references for this study but still have room for improvement in the following three aspects. First, in constructing the evaluation index system of EWP, the existing studies only consider the resource consumption on the input side but ignore the capital and labor factors that transform resource consumption into objective well-being levels. Second, studies have shown significant regional differences in EWP in China [34,36]. However, most of the studies on EWP are focused on the intuitive comparison of efficiency, and studies on the quantitative measurement of the degree of regional differences and the sources of regional differences are few. Finally, the studies on the factors influencing EWP are mainly based on regression models, revealing the overall influence of explanatory variables on EWP. However, they do not consider temporal and spatial factors, thus making the regression results possibly biased and unable to analyze the direction and magnitude of the influence of each explanatory variable on provinces’ EWP.

The marginal contributions of this study are as follows. First, this study comprehensively constructs an EWP evaluation system in China. Comprehensive well-being is characterized by economic, social, and environmental dimensions, and environmental pollution is characterized through wastewater, waste gas, industrial solid waste, and carbon emissions. The EWP of 30 Chinese provinces is measured quantitatively using the Super-SBM model. Second, China’s regional unbalance of EWP is measured and analyzed from four regions and eight regions using a combination of Theil index and Dagum Gini coefficient, revealing the sources of regional difference in EWP in China. Third, this study analyzes the drivers of EWP using a geographically and temporally weighted regression (GTWR) model innovatively, calculates the direction and magnitude of the influence of each explanatory variable on EWP, and reveals the spatial differences in the magnitude of their influence.

The construction of an evaluation index system for EWP breaks through the limitations of the traditional use of GDP to measure economic and social development status and provides a reference for other countries or regions to measure EWP levels. It also studies the regional differences in EWP, reveals the sources of their regional differences, explores the driving factors of EWP, and comprehensively examines the unbalanced status of regional EWP. Formulating strategies for differentiated improvement of regional EWP and promoting coordinated regional development are crucial.

## 3. Materials and Methods

### 3.1. Methods

#### 3.1.1. EWP Measurement: Super-SBM Model

The Super-SBM model proposed by Tone (2002) [37] considers both slack variables from a non-radial perspective and has the advantage that the efficiency values vary with the degrees of input and output slack. The model has now been widely used to measure EWP [32,34]. Therefore, this study adopted the super-efficiency SBM (Super-SBM) model considering undesirable outputs to measure the EWP of 30 provinces in China from 1997 to 2018. Suppose *n* decision units, *m* input elements, *s*_1_ desirable outputs, and *s*_2_ undesirable outputs are available, corresponding vectors are $x\in R_{m},y^{g}\in R_{s_{1}},y^{b}\in R_{s_{2}}$, matrix $X=(x_{ij})\in R_{m\times n},Y^{g}=(Y_{ij}^{g})\in R_{s_{1}\times n},Y^{b}=(Y_{ij}^{b})\in R_{s_{2}\times n}$ is defined, assuming $X>0,Y^{g}>0,Y^{b}>0$. The inputs and desirable outputs are freely disposed. The model can be expressed as follows:

(1) $$G=\{\left(x,\;y^{g},\;y^{b}\right)|x⩾X\lambda ,\;\;y^{g}⩽Y^{g}\lambda ,\;y^{b}=Y^{b}\lambda ,\;\sum_{i=1}^{n}\lambda =1,\;\lambda \geq 0$$

The super-SBM model is constructed by introducing undesirable outputs into the model, and the planning equation of the model is as follows:

(2) $$\begin{array}{c} \min\rho^{*}=\frac{\frac{1}{m}\sum_{i=1}^{m}\frac{s_{i}^{-}}{x_{i0}}}{\frac{1}{s_{1}+s_{2}}(\sum_{r=1}^{s_{1}}\frac{s_{r}^{g}}{y_{r0}^{g}}+\sum_{r=1}^{s_{2}}\frac{s_{r}^{b}}{y_{r0}^{b}})}\\ s.t.\;x_{0}=X\lambda +s^{-},y_{0}^{g}=Y^{g}\lambda -s^{g},y_{0}^{b}=Y^{b}\lambda +s^{b}\\ s^{-}⩾0,s^{g}⩾0,s^{b}⩾0,s^{g}⩾0,\lambda ⩾0 \end{array}$$

where $S^{-},S^{g}$, and $S^{b}$ denote the input, desired output, and undesired output slack variables, respectively; λ denotes the weight vector; $\rho^{*}$ denotes EWP, with larger values indicating higher levels of EWP. This study measures EWP based on Equation (2).

#### 3.1.2. Spatial Unbalanced Measurement: Dagum Gini Coefficient Decomposition

The Dagum Gini coefficient and its decomposition method proposed by Dagum (1997) [38] compensate for the shortcomings of traditional methods of measuring regional differences by decomposing the Gini coefficient by subgroups, effectively solving cross-over among samples. Therefore, this study used the Dagum Gini coefficient decomposition to measure the overall degree of differences quantitatively in EWP among the four and eight regions in China and reveal the sources that lead to differences in EWP. In this method, the overall Gini coefficient (*G*) is decomposed into intra-regional difference contribution (*G_w_*), inter-regional difference contribution (*G_nb_*), and hyper-variance density contribution (*G_t_*), and all three satisfy *G = G_w_ + G_nb_ + G_t_*. The overall Gini coefficient is calculated by Equation (3).

(3) $$G=\frac{\sum_{i=1}^{k}\sum_{m=1}^{k}\sum_{j=1}^{n_{i}}\sum_{r=1}^{n_{m}}\left|y_{ij}-y_{mr}\right|}{2\cdot n^{2}\cdot \mu }\;\;\;\mu_{m}\leq \cdots \leq \mu_{i}\leq \cdots \leq \mu_{k}$$

where *μ* is the mean value of EWP of each region and is ranked according to the mean value of EWP of each region. *n*, *k*, and $n_{i}\left(n_{m}\right)$ are the number of provinces, the number of regions, and the number of provinces in the *i*(*m*) region, respectively. $y_{ij}\left(y_{mr}\right)$ represents the EWP of the *j*(*r*) province in the *i*(*m*) region.

(4) $$G_{ii}=\frac{\sum_{j=1}^{n_{i}}\sum_{r=1}^{n_{i}}\left|y_{ij}-y_{ir}\right|}{2\cdot n_{i}^{2}\cdot \mu_{i}}$$

(5) $$G_{im}=\frac{\sum_{j=1}^{n_{i}}\sum_{r=1}^{n_{m}}\left|y_{ij}-y_{mr}\right|}{n_{i}\cdot n_{m}\cdot \left(\mu_{i}+\mu_{m}\right)}$$

(6) $$G_{gb}=G_{nb}+G_{t}$$

(7) $$G_{w}=\overset{k}{\underset{i=1}{\sum }}G_{ii}\cdot p_{i}\cdot s_{i}$$

(8) $$G_{nb}=\sum_{i=2}^{k}\sum_{m=1}^{i-1}G_{im}\cdot \left(p_{i}\cdot s_{m}+p_{m}\cdot s_{i}\right)\cdot D_{im}$$

(9) $$G_{t}=\sum_{i=2}^{k}\sum_{m=1}^{i-1}G_{im}\cdot \left(p_{i}\cdot s_{m}+p_{m}\cdot s_{i}\right)\cdot \left(1-D_{im}\right)$$

(10) $$D_{im}=\frac{d_{im}-p_{im}}{d_{im}+p_{im}}d_{im}=\int_{0}^{\infty }dF_{i}\left(y\right)\int_{0}^{y}\left(y-x\right)dF_{m}\left(x\right)$$

(11) $$p_{im}=\int_{0}^{\infty }dF_{m}\left(y\right)\int_{0}^{y}\left(y-x\right)dF_{i}\left(y\right)$$

(12) $$p_{i}=\frac{n_{i}}{n}s_{i}=\frac{n_{i}\cdot \mu_{i}}{n\cdot \mu }i=1,2,\cdots ,k$$

Equation (4) denotes the Gini coefficient for region *i*, and Equation (5) denotes the net inter-regional differences between regions *i* and *m*. $D_{im}$ denotes the relative impact of EWP between regions *i* and *m*. *F_i_* (*F_m_*) is the cumulative density distribution function for region *i*(*m*). $d_{im}$ denotes the difference in EWP between regions (i.e., the mathematical expectation of all sample values in regions *i* and *m* for which $y_{ij}-y_{mr}>0$ in the *i* and *m* regions), and $p_{im}$ is the mathematical expectation of the sum of all sample values of $y_{mr}-y_{ij}>0$ in the *i* and *m* regions.

#### 3.1.3. Analysis of Influencing Factors: GTWR

Unlike the traditional geographically weighted regression model that only considers the spatial dimension, geographically and temporally weighted regression (GTWR) incorporates the temporal dimension, thus providing an analytical basis for dealing with the “time–space” non-stationarity. Tian and Li (2020) apply the GTWR model to explore the factors influencing the ecological footprint of Zhejiang Province of China [39]. This study uses geographically and temporally weighted regression to analyze the impact factors of different spatial–temporal dimensions. The GTWR model [40,41] equation is as follows:

(13) $$EWP_{i}=\beta_{0}\left(u_{i},v_{i},t_{i}\right)+\sum_{k}\beta_{k}\left(u_{i},v_{i},t_{i}\right)X_{ik}+\varepsilon_{i}$$

where $EWP_{i}$ is the observed value of *EWP* in *i* region; $u_{i}$ and $v_{i}$ are the dimension and longitude of the *i* observation, respectively; $t_{i}$ is the time sequence in which *i* observation is made; $\left(u_{i},v_{i},t_{i}\right)$ is the spatial–temporal coordinate of *i* observation; $\beta_{0}\left(u_{i},v_{i},t_{i}\right)$ is the regression constant; $\beta_{k}\left(u_{i},v_{i},t_{i}\right)$ is the regression coefficient of the *k* independent variable; $X_{ik}$ is the value of the *k* independent variable at *i* point; $\varepsilon_{i}$ is the residual.

In existing studies, industrial structure (IS), degree of openness (FDI) [42], environmental regulation (ER) [2], and urbanization level (UR) [35] have been used to investigate efficiency. To examine the factors influencing EWP, we set the variables as follows: (1) UR. China’s urbanization rate had increased from 17.9 in 1978 to 59.58% in 2018. On the one hand, integrating education and medical resources during urbanization improves social well-being. By contrast, urbanization promotes the transfer of population from primary to secondary and tertiary industries to improve economic well-being [43]. However, on the other hand, urbanization leads to environmental pollution problems, such as air pollution and water quality deterioration, thus reducing environmental well-being [44]. UR is characterized by the number of the urban population in each province as a percentage of the total population in that area. (2) IS. The high input, high consumption, and high pollution characteristics of the secondary industry enhance people’s economic well-being while reducing environmental well-being [45]. IS is expressed as the share of the added value of the secondary industry in the regional gross domestic product (GDP). (3) ER. Increasing ER can discourage the development of pollution-intensive industries and improve people’s environmental well-being, but excessive ER may affect the development of regional economies. ER is characterized by the share of investment in environmental pollution control in GDP. (4) Degree of openness (FDI). The expansion of FDI will harm the environment of the host country and reduce environmental well-being [46]. However, at the same time, it will bring advanced technology and management experience, promote regional economic development, and improve economic well-being [47]. The degree of openness is characterized by foreign direct investment. (5) Marketization level (MI). Increasing MI can promote resource utilization. However, excessive marketization may lead to disorderly competition and have adverse effects on economic growth. The marketization index is used to characterize MI. (6) Water resource endowment (WR). The abundance of WR affects environmental well-being. If water resources are abundant in a region but underutilized, then EWP can also be harmed. Total water resources characterize water endowment. The correlation coefficients of the variables are presented in Table 2, and the results indicate no problem of multicollinearity among the variables.

### 3.2. Materials

#### 3.2.1. Measurement for EWP in China

**Figure 2:**
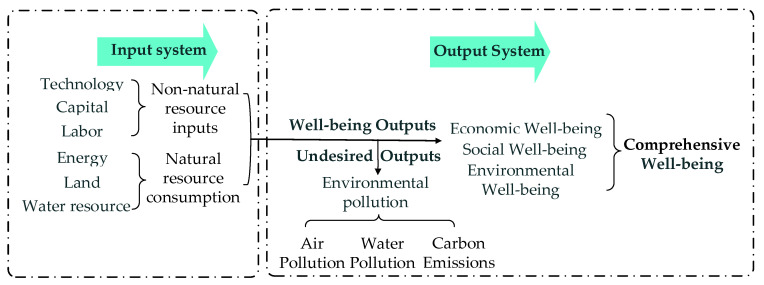
Ecological well-being performance measurement index system in China.

Figure 2 shows the measurement index system for studying the EWP of China’s provinces. The left input side is divided into two parts: natural resource inputs and non-natural resource inputs. Zhang et al. (2018) define EWP as the efficiency of the process of transforming ecological consumption into human well-being [30]. The human processing of resources into natural products that enhance human well-being requires technological, capital, and labor inputs as intermediate means [36], which are indispensable for the transformation from ecological consumption to human well-being. Income, education, and health cover economic and social dimensions, when extending EWP to the context of sustainable development, the whole process must be compatible with the environment. Reinhard et al. (2000) put environmental pollution as a cost in the production process on the input side when calculating environmental efficiency [48]. At the same time, this study argues that, in converting ecological consumption into well-being level, environmental pollution is a factor that reduces the comprehensive human well-being level. Its resource-like consumption that leads to environmental pollution has been put as a cost on the input side, and putting environmental pollution as an input again leads to double counting. Thus, this study corrects EWP by making environmental pollution-type indicators as undesired outputs. EWP is defined as the efficiency of transforming natural and non-natural resource consumption to a comprehensive well-being level that includes economic, social, and environmental well-being, corrected by environmental pollution.

#### 3.2.2. EWP Evaluation Index System

This study measures the EWP of 30 provinces in China from 1997 to 2018 using the Super-SBM model. Table 3 shows the evaluation index system of EWP in China’s provinces. Wang and Feng (2020) [34] and Yao et al. (2021) [3] measure the level of well-being output by using energy, land, and water consumption as inputs, ignoring the importance of technology, labor, and capital in integrated well-being transformation. Based on previous studies on EWP [36], this study adds to the input indicators characterizing technology, capital, and labor; expenditure on R&D per capita; investment in fixed asset per capita; employed persons. Among the output indicators, well-being output is divided into three dimensions of economic well-being, social well-being, and environmental well-being to reflect people’s comprehensive well-being output level in each province. Furthermore, environmental pollution, which harms the well-being output level, is included as a non-desired output in the evaluation index system of EWP. As the DEA has strict requirements on the number of input indicators. This study downscaled the four indicators characterizing environmental well-being output and the four categories of pollutants characterizing environmental pollution by principal global components to obtain the environmental well-being index and the environmental pollution index and thus control the number of input–output indicators and improve the model recognition.

Among the input indicators, expenditure on R&D per capita and investment in fixed asset per capita is calculated as stocks by the perpetual inventory method, which characterizes technology input and capital input. Taking investment in the fixed asset as an example, $K_{it},\;I_{it}$, and $\delta_{it}$ denote the capital stock, fixed asset investment, and capital depreciation rate of the *i* province, respectively, in period *t*. The formula for the perpetual inventory method is $K_{it}=\left(1-\delta_{it}\right)\cdot K_{i\left(t-1\right)}+I_{it}$. The base period capital stock is calculated as $K_{0}=I_{0}/\left(g_{i}+\delta \right)$, where $g_{i}$ is the geometric average growth rate of the fixed asset investment in province *i*. Expenditure on R&D and investment in fixed assets was converted to compare prices with 2000 as the base period before estimating the stock. The number of employed persons characterizes labor input. Energy, land, and water consumption are characterized by energy consumption per capita, area of built districts per capital, and water consumption per capita, respectively. Among the output indicators, GDP per capita is used to characterize the economic well-being output of each province, and was converted to constant prices in 2000 as the base period before calculating GDP per capita. The average years of education and the average life expectancy characterize the level of education and the level of health care of social well-being outputs, respectively. The formula uses the average years of education in Wang and Feng (2020) [34]. We used the three indicators characterizing technology, capital, and labor inputs to represent non-natural resource consumption. Meanwhile, energy, land, and water consumption represent natural resource consumption. The levels of economic development, educational development and health care, and environmental well-being are used to characterize comprehensive well-being, and four categories of pollutants are used to represent environmental pollution.

We performed descriptive statistics on all variables before calculating EWP and running the GTWR model. The results are presented in Table 4.

#### 3.2.3. Data Sources and Division of Regions

Data on expenditure on R&D by province are from the China Statistical Yearbook on Science and Technology: (1998–2019). Data on energy consumption by province are from the China Energy Statistical Yearbook (1998–2019). Data on the four indicators characterizing ecological and environmental well-being and the four categories of pollutants characterizing environmental pollution are from the China Environmental Statistical Yearbook (1998–2019). Data on average life expectancy are from the China Population and Employment Statistical Yearbook. Data on carbon emissions were obtained from the China Carbon Accounting Database (https://www.ceads.net.cn, accessed on 26 March 2021). Marketability index data are from the China Marketability Index Report. Data for other indicators are from the China Statistical Yearbook (1998–2019). Missing data for specific indicators in individual years are supplemented by internal and external interpolation.

The four major regions are the eastern, central, western, and northeastern regions. The eight regions are the northern coast, the northeastern, the eastern coast, the middle Yellow River, the southern coast, the northwest, the southwest, and the middle Yangtze River. The specific provinces included in the four major and eight regions are referred to as Ma et al. (2019) [49] and Wang et al. (2021) [50].

This study measured the EWP of 30 provinces in China using MaxDEA 6.0 software. Matlab R2015b (MathWorks, Natick, Massachusetts, United States) was used to calculate the Dagum Gini coefficients and their decomposition terms for the regions. ArcGIS 10.7 (Environmental Systems Research Institute, RedLands, United States) was used to calculate the magnitude and direction of the effects of each explanatory variable on EWP.

## 4. Results

### 4.1. Analysis of the Spatial and Temporal Evolution of EWP

#### 4.1.1. Time-Series Evolution of EWP in China

From Table 5, China’s EWP is characterized by a rise followed by a fluctuating decline from 1997 to 2018, and the EWP of the four regions as a whole, from high to low, is the eastern (1.182), western (1.060), central (1.036), and northeastern (1.028) regions. The eastern region’s EWP was higher than the national average in 1997–2018, and its efficiency is closest to the production frontier, which is consistent with Song and Mei’s (2021) [51] findings. By contrast, the central, western, and northeastern regions’ EWP differs slightly from Song and Mei’s findings, which may be due to model differences. However, the studies suggest that improving EWP in the western and northeastern regions is key to improving the overall level.

The eastern region’s EWP increased slightly from 1997 to 2018, the western region showed a fluctuating downward trend from 1997 to 2018, and the central and northeastern regions showed a clear upward trend in recent years. The western region’s EWP was ahead of the central and northeastern regions from 1997 to 2012, and the central and northeastern regions’ EWP surpassed that of the western region in 2013 and 2015, respectively. The central and northeastern regions have shown a clear upward trend in recent years, whereas the western region’s EWP has been characterized by an apparent decline in fluctuations after 2008. Therefore, special attention should be paid to lowering the western region’s EWP when formulating policies to improve regional EWP. In 2010, the EWP of the country and the central, western, and northeastern regions declined rapidly, mainly because Anhui, Xinjiang, and Jilin had different degrees of redundancy in labor, investment in fixed assets, and energy consumption, and economic and social well-being still has room for improvement. However, the central, western, and northeastern regions’ EWP tend to increase after 2010, indicating that the government has taken measures to control the declining trend.

In 1997, the eastern region’s EWP was the absolute leader, and the national, central, western, and northeastern regions were relatively close. Given that the absolute gap between regions has been increasing, especially in 2010, the EWP of the eastern region (1.209) was 1.28 times that of the northeastern region (0.940).

The EWP of the northern coast and southern coast is higher than that of other regions in 1997–2018, which is an important reason why the eastern region’s EWP is significantly higher than that of the other three regions. The eastern coastal region efficiency pulls down the EWP of the eastern region because of the drag of Jiangsu Province’s EWP. The average value of Jiangsu Province’s EWP is only 0.735 in 1997–2018, which is much lower than the national average. In the input–output slack results (results not given, available from the authors), the non-resource consumption indicators and energy consumption indicators of Jiangsu Province have excessive inputs in most years. At the same time, outputs and severe environmental pollution are observed to be simultaneously insufficient, making Jiangsu Province’s EWP pull down the EWP level of the eastern coastal region. The EWP of Southwest China and the middle Yellow River is in the middle position of the eight regions and close to the national average. The EWP of the Northwest, Middle Yangtze River, and Northeast regions is lower than the national average. The key to improving EWP in the western region lies in improving the four northwestern provinces’ EWP and improving EWP level in the central region lies in improving the middle Yangtze River’s EWP.

#### 4.1.2. Spatial Distribution Patterns of EWP in China

The spatial distribution pattern of EWP in China in 2018 can be analyzed from Table 5. In 2018, China’s top 10 EWPs were in Hainan, Beijing, Tianjin, Qinghai, Yunnan, Shanxi, Guangdong, Shanghai, Henan, and Hunan. Among them, five provinces were in the eastern region, three in the central region, and two in the western region. In the four major regions, the eastern region’s EWP value was the highest, followed by the northeastern, central, and western regions. In the eight regions, the southern coast had the highest EWP value, followed by the northern coast, the southwest, the middle Yellow River, the northeast, the middle Yangtze River, the eastern coast, and the northwest. The southern and northern coasts have apparent advantages in human social capital, progressive education, with less environmental pollution. The southwest region has a more impoverished industrial base but consumes fewer resources than other regions and is ahead of the national leader in environmental well-being. The eastern coastal region ranks relatively low among the eight regions because of the drag of Jiangsu Province’s EWP. The EWP of the south region is 1.081, higher than that of the north region at 1.063, with a small overall gap. At the provincial level, Hainan Province ranks first probably because of its industrial development focus on ecotourism, real estate, and sustainable industries that focus on reducing pollution [32]. Beijing ranked second because of its current ecological and environmental protection policies, environmentally friendly industries, and leading medical and educational standards. Hou et al. (2020) [10] also find that Beijing and Hainan are ahead of other provinces regarding EWP. They conclude that both perform well in terms of environmental protection.

### 4.2. Spatially Unbalanced Analysis of EWP in China

The analysis of EWP in the previous section of this paper identified spatially unbalanced features. We adopted the Theil index and the Dagum Gini coefficient to measure the overall differences in China’s EWP quantitatively and decompose the overall differences through the Dagum Gini coefficient decomposition method and thus reveal more precisely the size of the relative gap in China’s EWP and its sources in the next section.

#### 4.2.1. Spatial Unbalance of EWP in China

**Figure 3:**
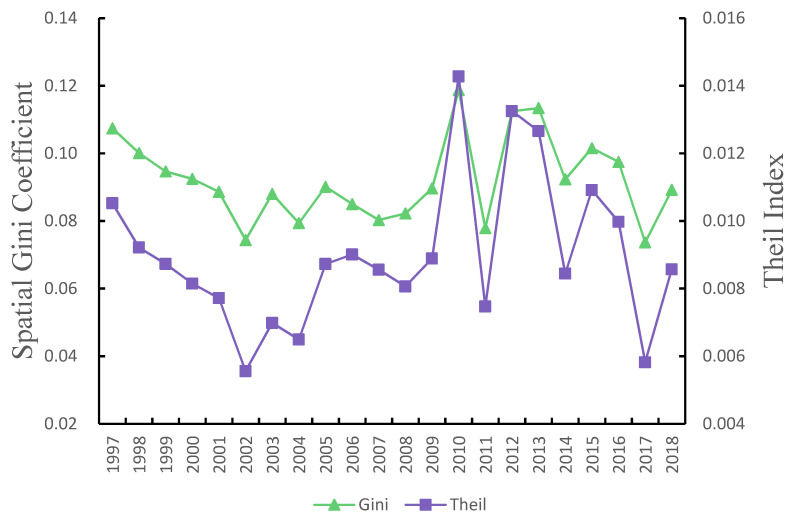
Spatial Gini coefficient and Theil index of ecological well-being performance in China.

Figure 3 shows the spatial Gini coefficient and Theil index of China’s EWP decreased from 1997 to 2018, indicating that the inter-provincial differences decreased from 1997 to 2018. The trends of the Gini coefficient and the Theil index of EWP in China are the same, with an overall characteristic of “falling–rising–fluctuating and falling,” indicating that the overall difference in EWP in China tends to decrease. Specifically, from 1997 to 2002, the Gini coefficient and the Theil index of EWP in China continued to decline, indicating that China’s EWP’s overall differences continued to decrease during this period. From 2003 to 2010, the Gini coefficient and the Theil index of EWP continued to increase, especially from 2009 to 2010, indicating that the unbalanced EWP continued to deteriorate in this period. After 2010, the Gini coefficient and the Theil index of EWP shows a fluctuating downward trend, and the problem of unbalanced EWP has been alleviated. During the 12th Five-Year Plan period, China proposed the overall regional development strategy and the primary functional area strategy and insisted on reducing regional disparities because of regional development strategies and policies.

#### 4.2.2. Unbalanced Analysis of EWP in Four Regions

The spatial unbalance of China’s EWP was analyzed from the four major regions (Table 6). As shown in Table 6, the differences in EWP among these regions gradually changed from differences among provinces within the region to differences between regions. In terms of the Gini coefficient, the intra-regional Gini coefficient fluctuates less, and hyper-variance density decreased significantly in 1997–2018, and inter-regional difference is more stable in 1997–2008 but fluctuates significantly after that. Before 2009, the intra-regional difference, inter-regional difference, and hyper-variance density of the four regions’ EWP are 0.026, 0.026, and 0.036, respectively, and hyper-variance density was the primary source of the overall difference. After 2009, the intra-regional difference, inter-regional difference, and hyper-variance density of the four major regions were 0.028, 0.039, and 0.031, respectively, with the inter-regional difference being the primary source of the overall difference. From the perspective of contribution rate, the contribution rate of intra-regional difference was higher than the contribution rate of inter-regional difference in 1997–2000, indicating that the differences in China’s EWP in this period were mainly manifested as intra-regional province-to-province differences. In 2001, the contribution rate of inter-regional differences exceeded the contribution rate of intra-regional differences, indicating that the differences in EWP began to change from differences among provinces within regions to differences among the four regions. After 2001, the contribution rate of intra-regional differences continued to decline, the inter-regional differences fluctuated and rose, and the differences between regions became the primary source of EWP in China.

The east (0.131) had the highest Gini coefficients intra-regional, followed by the west (0.068), central (0.061), and the northeast (0.021). The most significant regional differences in the east’s EWP are mainly because of high-value areas of EWP in the region, such as Hainan, Beijing, and Shanghai. In terms of the evolution of intra-regional differences, the Gini coefficient in the eastern region continued to decline after 2010, indicating that the regional imbalance in the eastern region gradually improved, and the Gini coefficient in the central and northeastern regions tend to increase slightly in recent years. Measures should be taken to avoid a continuous deterioration of this situation. The Gini coefficient of the western region has been changing in recent years, indicating that the regions have actively adjusted their strategies. However, attention should be paid to the mechanism of the effect of strategy implementation on the regional unbalance situation, and the combination of the two aspects should improve EWP within the region while reducing regional differences.

#### 4.2.3. Unbalanced Analysis of EWP in Eight Regions

This study quantitatively measured the Gini coefficient of eight regions’ EWP in China. Table 7 shows that inter-regional differences are the most crucial source of eight regions’ EWP in China. From 1997 to 2018, the mean values of intra-regional differences, inter-regional differences, and hyper-variance density of eight regions in China were 0.008, 0.062, and 0.022, respectively, and inter-regional differences were significantly more significant than intra-regional differences and hyper-variance density. Regarding the trend of contribution rate changes, in 1997–2018, the contribution rate of intra-regional difference decreases from 10.27% to 9.08%, and the contribution rate of inter-regional difference increases from 53.44% to 68.34%. Intra-regional difference kept decreasing, and inter-regional difference kept increasing, resulting in the primary source of EWP in China being the inter-regional difference, consistent with the results calculated from four regional divisions.

In terms of the Gini coefficient of EWP within the eight regions of China, the southern coast (0.147) has the highest, followed by the eastern coast (0.089), the northwest (0.088), the northern coast (0.073), the middle Yangtze River (0.067), the southwest (0.052), the northeast (0.021), and the middle Yellow River (0.015), with average annual growth rates of −0.80%, −0.17%, −0.07%, 1.61%, −3.83%, −6.98%, 0.02%, and 3.99%, respectively. In terms of change trends, although the average annual growth rate of the Gini coefficient of the southern coast is only −0.80%, the intra-regional differences have shown a continuous decreasing trend since 2007, indicating that the intra-regional differences in EWP have decreased. The Gini coefficient of the eastern coast fluctuated more from 1997 to 2018. The Gini coefficient of the northwest region decreased significantly until 2008. After that, it showed an alternating “up–down” trend and no convergence in EWP. Although the Gini coefficient of the northern coast has a decreasing trend in recent years, it is generally increasing from 1997 to 2018, and the problem of spatial imbalance has not improved. The Gini coefficients of the middle Yangtze River and the southwest region show a fluctuating downward trend from 1997 to 2018, and the EWP in the region converges significantly. The Gini coefficient of the northeast region in 2018 did not increase significantly compared with that of 1997, and the overall regional differences were minor. The Gini coefficient of the middle Yellow River was at the back of the eight regions in 1997–2018, with coordinated EWP within the region.

In 1997–2018, the overall spatial differences in China’s EWP narrowed, and the regional unbalance improved. The widening of inter-regional differences is the main reason for the overall differences among the four major regions and the eight regions. From the four major regions, inter-provincial differences in EWP are the largest in the eastern region, and the three northeastern provinces are relatively balanced. From the eight regions, the spatial unbalance of EWP is more significant in the southern coastal region. The convergence of the southwest region’s EWP is the fastest.

### 4.3. Analysis of the Actors Influencing EWP in China

We applied the GTWR model to examine the effect of each explanatory variable on EWP. We can examine the changes in the magnitude of the effect of each variable on EWP over time. Thus, we can provide a reference for the targeted development of strategies to enhance EWP. We used the GTWR model to examine the direction and magnitude of the effects of UR, IS, ER, FDI, MI, and WR on EWP.

Table 8 shows the parameters associated with GTWR. The value of AICc is −2196.1, which indicates that this GTWR model is effective. Regarding the goodness of fit, R^2^ is close to 0.65 with the adjusted R^2^, indicating that GTWR can measure the effect of the explanatory variables on the dependent variable better. Table 9 shows the results of GTWR model in 1997 and 2018.

#### 4.3.1. Impact of Urbanization Level on EWP

The contribution of urbanization to EWP rises and then falls. The average impact coefficient for the 30 provinces was 0.0947 in 1997 and began to fall after rising to 0.1054 in 2013, and this value fell to 0.0994 in 2018. This overall change suggests that the contribution of urbanization to EWP has begun to weaken.

The high impact of urbanization on EWP is concentrated in the eastern coast, especially in the middle Yangtze River, with Shanghai as the core. Furthermore, urbanization also has a significant positive impact on Qinghai, Gansu, and Hainan provinces. Most of the low-impact regions are located in the northeast and southwest regions. This result reflects that the contribution of urbanization to EWP is more evident in the east coast region, whereas the contribution of urbanization to EWP is weaker in the northeast and southwest regions.

#### 4.3.2. Impact of Industrial Structure on EWP

Industrial structure has a significant negative impact on EWP. Although the average impact coefficient of industrial structure on EWP increased from −0.1062 in 1997 to −0.089 in 2018, it still negatively impacted EWP. In recent years, with the increase of income, people have had higher demands on environmental quality. The industrial structure affects the improvement of EWP because of the contradiction between the rough development characteristics of the secondary industry and people’s high demand for environmental quality.

The impact of industrial structure on EWP has a significant spatial correlation. Table 9 shows that the regions where the hindering effect of industrial structure on EWP is significant are distributed south of the Yangtze River, especially in Guangdong, Guangxi, and Yunnan. The degree of hindrance gradually decreases as it extends northward.

#### 4.3.3. Impact of Environmental Regulation on EWP

Environmental regulation on EWP shifted from a positive facilitative effect to a negative hindering effect. The average coefficient of the impact of environmental regulation on EWP decreases from 0.0112 in 1997 to −0.0071 in 2018. The most significant decrease in the impact coefficient from 1997 to 2018 is mainly in the less economically developed western regions, such as Qinghai, Gansu, and Sichuan provinces. The hindering effect of environmental regulations on EWP in these areas may be that overly stringent environmental regulations hinder the already underdeveloped local economies and impede the enhancement of economic well-being, which in turn has a hindering effect on EWP [52].

#### 4.3.4. Impact of the Degree of Openness on EWP

The degree of openness has a suppressive effect on EWP in the eastern and central regions. In contrast, it has a facilitating effect on the western and northeastern regions, and the effect of degree of openness on EWP shows apparent heterogeneity. The coefficient of the impact of the degree of openness on EWP in the eastern region is negative. The coefficient of the impact in the central region is also negative but less than that in the eastern region. The coefficient of the impact in the western region is positive, which indicates that the “pollution paradise” hypothesis is not established in the western region.

#### 4.3.5. Impact of the Level of Marketization on EWP

The effect of marketization level on EWP is closely related to urban agglomerations or economic zones. The level of marketization in the middle Yangtze River city cluster and Beijing-Tianjin-Hebei city cluster significantly and positively affects EWP. The development and improvement of urban agglomerations enable inter-city resources to complement each other’s strengths, break down local administrative barriers, and promote economic and social development [53]. Except for the middle Yangtze River urban agglomeration and Beijing-Tianjin-Hebei urban agglomeration, the level of marketization in other regions still has a suppressive effect on EWP.

#### 4.3.6. Impact of Water Resources on EWP

The high-impact areas of water resources on EWP are concentrated in the middle Yangtze River and the east coast regions, such as Shanghai, Zhejiang, and Fujian provinces. These regions have high technological innovation capacity and high efficiency in the use of water resources. In contrast, although water resources are abundant in the southwest of China, the contradiction between supply and demand and the pollution of water resources make water resources in these regions harm EWP [54].

## 5. Discussion

The GDP measure of economic development is a standard and valid indicator, but it has been used unilaterally as a more general measure of well-being [55,56]. The single-minded pursuit of GDP growth does not lead to sustained improvements in human well-being, and the rate of improvement in human well-being begins to stagnate or decrease when economic growth reaches a certain point [57,58]. Academics have been searching for indicators that can accurately and comprehensively measure human well-being, and the HDI, the Real Progress Index, and the Happy Planet Index have been proposed one after another. However, researchers’ perspectives and purposes differ, and the specific indicators proposed have different focuses. Economic, social, and environmental factors significantly affect people’s well-being, and constructing a well-being indicator evaluation system that covers economic, social, and environmental dimensions to measure a country or region’s well-being development level is more comprehensive. EWP is a multidimensional concept involving economic, social, and environmental factors consistent with sustainable development. This study constructed a performance evaluation index system that can characterize economic, social, and environmental well-being. EWP measures the efficiency of well-being transformation in a country or region.

The measurement of EWP in 30 provinces by the Super-SBM model revealed that the level of economic development is not a decisive factor in determining the level of ecological well-being. Bian et al.’s article verified the same conclusion, justifying the inclusion of social and environmental well-being in the EWP evaluation system in this study [32]. The existence of a “welfare threshold” proves that increasing inputs does not promote sustainable growth in well-being. For example, the excessive demand for nature is causing the United States to suffer an ecological deficit, which inevitably impacts the improvement of people’s well-being [27].

EWP is characterized by significant regional unbalance, and studies have found that inter-regional differences are the primary source of overall differences [34] and reducing inter-regional differences has become a key consideration for current policy formulation. Breaking through the administrative barriers between regions and building inter-regional cooperation mechanisms are worthy of crucial exploration. Differences in resource endowments are the main reason for the inconsistent direction of industrial development in each region. Making full use of the region’s resource endowments, strengthening the interaction between institutions, such as industry, university, and other institutes [59], forming comparative advantages related to the region’s characteristic resources by taking advantage of the situation, and putting the concept of regional innovation system into practice are important ways to reduce inter-regional differences in EWP.

The purpose of high-quality economic development is to meet the growing needs of the people for a better life [5] in terms of income, education, health care, the environment, poverty, and other aspects [60]. High-quality economic development meets the requirements of sustainable development and the requirement to improve people’s well-being. High-quality economic development is indeed a viable path to achieve improved EWP. On the one hand, it takes advantage of innovation-driven economic growth to promote the efficient conversion of resources and low entropy emissions. On the other hand, it takes full advantage of big data to explore the needs that can improve people’s quality of life. Furthermore, differentiated strategies to improve EWP need to be developed for different regions. For the northern coastal regions, Beijing, Tianjin, and Hebei’s integrated development should continue to be strengthened, with emphasis on increasing environmental management. Attention should be paid to the environmental problems brought by urbanization for the northeastern region while attracting foreign investment. For the eastern coastal regions, controlling the scale of foreign direct investment is the key to improving regional EWP. For the middle Yellow River, the middle Yangtze River, and the southern coast, the key to improving EWP lies in adjusting the industrial structure, promoting the modernization of manufacturing industries and services, breaking down regional administrative barriers, and promoting coordinated development linkages among cities. For the northwest region, attracting foreign investment, playing the secondary industry’s role in promoting EWP, raising the level of urbanization, and improving people’s overall well-being are urgently needed. For the southwest region, improving the efficiency of water resources utilization is necessary.

## 6. Conclusions

This study used the Super-SBM model, Dagum Gini coefficient decomposition, and GTWR methods to investigate EWP, regional differences, and influencing factors in China. The EWP evaluation system constructed in this study is comprehensive and objective and involves various economic and social development aspects. The measurement of EWP in China can provide theoretical support for policy formulation, accurately grasp the current situation of EWP in China, and lay the foundation for formulating differentiated strategies to improve EWP. We analyzed the regional differences in EWP in China and discussed how to promote coordinated regional development. In this research, the factors influencing EWP were analyzed, and possible suggestions for improving regional EWP were provided. However, the study has some limitations, as the well-being indicators selected are all objective. At the same time, subjective well-being indicators are also important factors influencing well-being. Therefore, the integration of objective and subjective well-being indicators and the inclusion of inequality and poverty into the EWP evaluation index system are two of the directions for future research on EWP. Another research direction is the use of system dynamics to explore EWP in depth. The findings are as follows.

First, the EWP of 30 provinces declined slightly from 1997 to 2018. However, the overall degree of difference in EWP has also decreased, and the problem of regional unbalance has improved. China’s EWP shows prominent spatially unbalanced characteristics, with the highest EWP in the east, the lowest in the northeast, and intermediate in the central and western regions. Among the eight regions, the southern coast is far ahead of other regions, whereas the EWP of the northwest region is at the bottom of the eight regions.

Second, inter-regional differences are the primary source of regional differences in EWP in China. The internal differences in EWP of the four regions and the eight regions in China have been decreasing from 1997 to 2018, and the contribution of inter-regional differences to the overall differences has been increasing. The key to narrowing the development gap of EWP in China and promoting coordinated regional development in the future lies in controlling and narrowing inter-regional differences.

Third, urbanization has a significant positive effect on improving EWP in China, with significant heterogeneity in the effects of the degree of openness, industrial structure, environmental regulation, market level, and water endowment on EWP in different regions.

## Figures and Tables

**Table 1:** Studies related to ecological well-being performance.

Category	Method	Model	Author	Objective Area
Ecological well-being performance evaluation	Ratio method	The ratio of happy life index to Ecological Footprint	Abdallah et al. (2009) [29]	143 countries
		The ratio of HDI to Ecological Footprint	Zhang et al. (2018) [30]	82 countries
	Parametric method	Stochastic Frontier Analysis (SFA)	Dietz et al. (2009) [31]	135 countries
	Non-parametric methods	Super-SBM	Bian et al. (2020) [32]	30 provinces in China
		Super-SBM	Yao et al. (2021) [3]	30 provinces in China
		Data Envelopment Analysis (DEA)	Ibrahim et al. (2021) [33]	sub-Saharan African countries
Regional differences on EWP		Theil	Wang and Feng (2020) [34]	30 provinces in China
		Convergence analysis method	Li et al. (2020) [1]	30 provinces in China
Factors influencing EWP		Panel data models	Zhang et al. (2020) [35]	90 countries

**Table 2:** Correlation coefficients between the variables.

Variable	Description	UR	IS	ER	FDI	MI	WR
UR	Urbanization level/%	1	−0.045	0.215 ***	0.563 ***	0.61 ***	−0.434 ***
IS	Industrial structure/%	−0.045	1	0.13 ***	0.171 ***	0.055	0.028
ER	Environmental regulation/%	0.215 ***	0.13 ***	1	−0.137 ***	−0.108 ***	−0.39 ***
FDI	Degree of openness (logarithm)	0.563 ***	0.171 ***	0.137 ***	1	0.813 ***	0.018
MI	Level of marketization (logarithm)	0.61 ***	0.055	−0.108 ***	0.813 ***	1	−0.083 **
WR	Water resources (logarithm)	−0.434 ***	0.028	−0.39 ***	0.018	−0.083 **	1

Note: ***, ** represent significant levels of 1% and 5%, respectively.

**Table 3:** Evaluation index system for ecological well-being performance.

Category	1st Tier Indicators		2nd Tier Indicators	3rd Tier Indicators
Input indicators	Non-natural resource inputs		Technology	Expenditure on R&D per capita
			Capital	Investment in fixed asset per capita
			Labor	Employed persons
	Natural resource consumption		Energy	Energy consumption per capita
			Land	Area of built districts per capital
			Water resource	Water consumption per capita
Output indicators	Desirable outputs: comprehensive well-being	Economic well-being	Economic	Per capita GDP
		Social well-being	Education	Average years of education
			Health care	Average life expectancy
		Environmental well-being	Environmental well-being level	Forest coverage
				The proportion of nature reserves in the area of the jurisdiction
				Greening coverage of the built area
				Park area per capita
	Undesirable outputs: environmental pollution	Environmental Pollution Index	Wastewater	Wastewater discharge per capita
			Exhaust gas	Waste gas emissions per capita
			Solid waste	Industrial solid waste per capita
			Carbon emissions	Carbon emissions per capita

**Table 4:** Descriptive statistics of input and output indicators and explanatory variables.

Category	Variable	Obs	Min	Max	Mean	Std. Dev.
Input and output indicators	Technology	660	28.001	34,635.826	1891.087	3750.040
	Capital	660	2243.591	448,151.708	83,223.992	79,729.157
	Labor	660	254.800	6767.000	2482.318	1656.835
	Energy	660	0.484	10.320	2.657	1.609
	Land	660	6.942	81.051	30.393	14.733
	Water resource	660	161.200	2738.895	515.241	438.217
	Economic	660	2153.116	96,286.977	20,807.850	16,477.114
	Education	660	4.690	12.880	8.409	1.185
	Health care	660	63.118	82.745	74.266	3.514
	Environmental well-being level	660	5.361	32.374	19.738	5.866
	Environmental Pollution Index	660	2.056	20.510	6.437	3.130
Explanatory variables	UR	660	14.039	89.600	47.971	15.989
	IS	660	18.630	61.500	45.682	7.834
	ER	660	0.061	4.231	1.242	0.709
	FDI (logarithm)	660	0.746	3.179	1.965	0.758
	MI (logarithm)	660	0.111	1.069	0.745	0.156
	WR (logarithm)	660	0.925	3.470	2.596	0.632

**Table 5:** Ecological well-being performance of 30 provinces, four regions and eight regions in China: 1997–2018.

Region	1997	1998	1999	2000	2001	2002	2003	2004	2005	2006	2007
Anhui	1.004	1.017	1.017	1.032	1.005	1.022	1.045	1.075	1.077	1.055	1.050
Beijing	1.106	1.115	1.124	1.117	1.125	1.218	1.263	1.276	1.338	1.349	1.333
Fujian	1.222	1.213	1.205	1.195	1.133	1.132	1.120	1.116	1.122	1.101	1.097
Gansu	0.560	0.570	0.589	0.607	0.663	1.005	1.012	1.013	1.012	1.011	1.004
Guangdong	1.042	1.030	1.027	1.034	1.037	1.029	1.041	1.043	1.042	1.074	1.060
Guangxi	1.007	1.006	1.006	1.028	1.020	1.033	1.062	1.089	1.090	1.106	1.095
Guizhou	1.606	1.453	1.492	1.603	1.490	1.515	1.446	1.374	1.343	1.279	1.315
Hainan	1.945	1.882	1.987	1.874	1.836	1.751	1.731	1.866	2.144	2.255	2.258
Hebei	1.041	1.059	1.006	1.002	1.007	1.005	1.018	1.022	1.020	1.022	1.019
Henan	1.090	1.100	1.098	1.118	1.091	1.111	1.128	1.131	1.147	1.116	1.128
Heilongjiang	1.022	1.018	1.016	1.018	1.049	1.035	1.038	1.048	1.075	1.067	1.065
Hubei	0.711	0.735	0.700	0.691	0.624	0.648	0.692	0.777	0.811	0.874	1.005
Hunan	1.028	1.026	1.024	1.036	1.004	1.013	1.008	1.021	1.026	1.043	1.052
Jilin	1.112	1.126	1.119	1.076	1.055	1.057	1.052	1.053	1.038	1.019	1.016
Jiangsu	0.676	0.686	1.001	1.002	1.000	1.000	0.644	0.645	0.682	0.668	0.696
Jiangxi	1.319	1.401	1.326	1.237	1.279	1.172	1.154	1.097	1.106	1.064	1.071
Liaoning	1.061	1.063	1.051	1.058	1.069	1.057	1.062	1.046	1.036	1.020	1.016
Inner Mongolia	1.143	1.127	1.125	1.147	1.119	1.099	1.068	1.048	1.037	1.036	1.038
Ningxia	1.100	1.103	1.058	1.058	1.058	1.057	1.033	1.025	1.022	1.025	1.026
Qinghai	1.332	1.282	1.298	1.258	1.094	1.081	1.092	1.063	1.084	1.083	1.089
Shandong	1.051	1.045	1.051	1.051	1.057	1.061	1.053	1.046	1.045	1.048	1.042
Shanxi	1.086	1.090	1.128	1.143	1.137	1.141	1.126	1.148	1.178	1.182	1.167
Shaanxi	1.089	1.076	1.168	1.171	1.151	1.106	1.141	1.132	1.123	1.111	1.108
Shanghai	1.159	1.124	1.119	1.113	1.117	1.151	1.174	1.107	1.107	1.104	1.104
Sichuan	1.015	1.039	1.041	1.017	1.017	1.001	0.801	1.010	0.829	1.016	1.016
Tianjin	1.340	1.327	1.293	1.331	1.418	1.378	1.384	1.369	1.340	1.359	1.345
Xinjiang	1.039	1.027	1.040	1.043	1.011	1.023	1.017	1.010	1.001	1.006	1.007
Yunnan	1.113	1.078	1.093	1.101	1.116	1.107	1.120	1.125	1.090	1.109	1.091
Zhejiang	1.034	1.035	1.040	1.035	1.036	1.034	1.032	1.031	1.025	1.036	1.042
Chongqing	1.163	1.137	1.146	1.182	1.145	1.160	1.177	1.142	1.055	1.057	1.043
East	1.161	1.152	1.185	1.175	1.177	1.176	1.146	1.152	1.186	1.202	1.200
Northeast	1.065	1.069	1.062	1.051	1.058	1.050	1.051	1.049	1.050	1.035	1.032
Central	1.040	1.061	1.049	1.043	1.023	1.018	1.025	1.042	1.058	1.056	1.079
West	1.106	1.082	1.096	1.110	1.080	1.108	1.088	1.094	1.062	1.076	1.076
Northern coast	1.135	1.137	1.118	1.125	1.152	1.165	1.180	1.178	1.186	1.194	1.185
Eastern coast	0.956	0.948	1.053	1.050	1.051	1.062	0.950	0.928	0.938	0.936	0.947
Southern coast	1.403	1.375	1.406	1.367	1.336	1.304	1.297	1.341	1.436	1.477	1.472
Northeast	1.065	1.069	1.062	1.051	1.058	1.050	1.051	1.049	1.050	1.035	1.032
Middle Yellow River	1.102	1.098	1.130	1.145	1.125	1.114	1.115	1.115	1.121	1.111	1.110
Middle Yangtze River	1.016	1.045	1.017	0.999	0.978	0.964	0.975	0.993	1.005	1.009	1.045
Northwest	1.008	0.995	0.996	0.992	0.957	1.042	1.038	1.028	1.030	1.031	1.031
Southwest	1.181	1.142	1.156	1.186	1.158	1.163	1.121	1.148	1.081	1.113	1.112
**Region**	**2008**	**2009**	**2010**	**2011**	**2012**	**2013**	**2014**	**2015**	**2016**	**2017**	**2018**
Anhui	1.023	1.011	0.664	1.001	0.736	0.719	1.006	1.002	1.004	1.002	1.007
Beijing	1.368	1.363	1.446	1.492	1.561	1.546	1.557	1.544	1.461	1.424	1.430
Fujian	1.093	1.090	1.081	1.077	1.074	1.080	1.081	1.078	1.074	1.076	1.077
Gansu	1.000	0.694	1.005	1.001	1.001	0.725	0.730	0.744	0.766	1.013	1.027
Guangdong	1.071	1.077	1.091	1.096	1.108	1.112	1.122	1.128	1.130	1.130	1.125
Guangxi	1.072	1.073	1.020	1.023	1.014	1.019	1.024	1.026	1.022	1.019	1.020
Guizhou	1.339	1.278	1.248	1.244	1.207	1.199	1.180	1.158	1.146	1.107	1.089
Hainan	2.149	2.116	2.229	2.078	2.082	1.952	1.969	1.935	1.875	1.842	1.802
Hebei	1.024	1.031	1.038	1.037	1.047	1.051	1.044	1.049	1.046	1.047	1.043
Henan	1.114	1.100	1.092	1.097	1.082	1.081	1.114	1.101	1.111	1.110	1.103
Heilongjiang	1.064	1.057	1.039	1.044	1.042	1.047	1.053	1.060	1.073	1.083	1.091
Hubei	0.906	0.874	0.831	1.000	1.003	1.003	0.884	0.917	0.893	0.866	0.838
Hunan	1.054	1.053	1.064	1.056	1.049	1.067	1.079	1.092	1.104	1.110	1.099
Jilin	1.020	1.005	0.772	1.001	0.740	0.793	0.859	1.001	1.007	1.004	1.002
Jiangsu	0.694	0.689	0.678	0.706	0.684	0.691	0.680	0.673	0.662	0.655	0.658
Jiangxi	1.058	1.048	1.042	1.042	1.054	1.044	1.028	1.022	1.018	1.018	1.018
Liaoning	1.011	1.016	1.010	1.017	1.029	1.035	1.031	1.029	1.021	1.024	1.033
Inner Mongolia	1.043	1.042	1.038	1.046	1.046	1.036	1.035	1.040	1.049	1.038	1.041
Ningxia	1.037	1.025	1.028	1.016	1.019	1.023	1.020	1.038	1.039	1.035	1.017
Qinghai	1.093	1.105	1.115	1.125	1.172	1.142	1.166	1.159	1.146	1.162	1.192
Shandong	1.038	1.038	1.027	1.023	1.025	1.027	1.022	1.027	1.031	1.037	1.037
Shanxi	1.179	1.162	1.147	1.125	1.095	1.075	1.082	1.090	1.089	1.115	1.145
Shaanxi	1.103	1.103	1.106	1.098	1.088	1.075	1.061	1.067	1.067	1.043	1.039
Shanghai	1.118	1.112	1.105	1.071	1.071	1.069	1.093	1.119	1.137	1.148	1.116
Sichuan	1.022	1.027	1.031	1.068	1.092	1.095	1.082	1.080	1.094	1.081	1.089
Tianjin	1.358	1.338	1.348	1.350	1.357	1.336	1.334	1.314	1.300	1.296	1.267
Xinjiang	1.004	1.004	0.481	1.005	0.427	0.432	1.001	0.444	0.453	1.001	0.476
Yunnan	1.080	1.083	1.085	1.090	1.100	1.125	1.151	1.122	1.108	1.133	1.152
Zhejiang	1.038	1.065	1.057	1.053	1.053	1.039	1.039	1.038	1.038	1.043	1.046
Chongqing	1.031	1.021	1.023	1.030	1.052	1.053	1.060	1.078	1.080	1.082	1.082
East	1.195	1.192	1.210	1.198	1.206	1.190	1.194	1.190	1.175	1.170	1.160
Northeast	1.032	1.026	0.941	1.021	0.937	0.958	0.981	1.030	1.033	1.037	1.042
Central	1.056	1.041	0.973	1.054	1.003	0.998	1.032	1.037	1.036	1.037	1.035
West	1.075	1.041	1.016	1.068	1.020	0.993	1.046	0.996	0.997	1.065	1.020
Northern coast	1.197	1.192	1.215	1.226	1.247	1.240	1.239	1.233	1.209	1.201	1.194
Eastern coast	0.950	0.955	0.947	0.943	0.936	0.933	0.937	0.943	0.946	0.949	0.940
Southern coast	1.438	1.428	1.467	1.417	1.421	1.381	1.390	1.380	1.360	1.349	1.334
Northeast	1.032	1.026	0.941	1.021	0.937	0.958	0.981	1.030	1.033	1.037	1.042
Middle Yellow River	1.110	1.102	1.096	1.091	1.078	1.067	1.073	1.074	1.079	1.077	1.082
Middle Yangtze River	1.010	0.997	0.900	1.025	0.960	0.958	0.999	1.008	1.005	0.999	0.990
Northwest	1.033	0.957	0.907	1.037	0.905	0.830	0.979	0.846	0.851	1.053	0.928
Southwest	1.109	1.096	1.081	1.091	1.093	1.098	1.099	1.093	1.090	1.084	1.086

**Table 6:** Gini coefficients and contribution rates of the four regions.

Year	Nationwide			Gini Coefficient by Region				Contribution Rate (%)		
	G*_w_*	G*_nb_*	G*_t_*	East	Central	West	Northeast	G*_w_*	G*_nb_*	G*_t_*
1997	0.033	0.023	0.052	0.128	0.090	0.108	0.019	30.66%	20.99%	48.35%
1998	0.030	0.018	0.052	0.122	0.095	0.092	0.022	30.27%	17.68%	52.05%
1999	0.028	0.026	0.040	0.102	0.094	0.096	0.022	30.07%	27.57%	42.36%
2000	0.028	0.025	0.039	0.096	0.084	0.101	0.012	30.29%	27.45%	42.26%
2001	0.026	0.029	0.034	0.095	0.102	0.082	0.004	29.31%	32.54%	38.14%
2002	0.021	0.029	0.024	0.091	0.084	0.054	0.005	28.51%	39.38%	32.11%
2003	0.026	0.023	0.039	0.122	0.074	0.068	0.005	29.73%	25.80%	44.47%
2004	0.023	0.021	0.035	0.130	0.059	0.045	0.001	29.37%	27.04%	43.59%
2005	0.027	0.027	0.037	0.145	0.058	0.054	0.008	29.69%	29.51%	40.81%
2006	0.025	0.031	0.030	0.153	0.047	0.035	0.010	29.18%	36.05%	34.77%
2007	0.024	0.028	0.028	0.150	0.027	0.036	0.011	29.75%	35.29%	34.96%
2008	0.024	0.030	0.028	0.145	0.043	0.037	0.011	29.28%	36.16%	34.56%
2009	0.027	0.032	0.031	0.141	0.046	0.059	0.011	29.69%	35.67%	34.64%
2010	0.033	0.052	0.034	0.154	0.092	0.076	0.063	27.84%	43.51%	28.64%
2011	0.023	0.032	0.023	0.145	0.024	0.033	0.010	29.17%	40.67%	30.17%
2012	0.032	0.048	0.033	0.150	0.056	0.082	0.072	28.39%	42.25%	29.36%
2013	0.033	0.045	0.036	0.140	0.057	0.100	0.059	28.97%	39.33%	31.70%
2014	0.026	0.037	0.029	0.143	0.038	0.054	0.044	28.56%	40.30%	31.14%
2015	0.030	0.041	0.030	0.140	0.034	0.094	0.013	29.88%	40.16%	29.96%
2016	0.029	0.037	0.031	0.132	0.039	0.089	0.014	29.79%	38.42%	31.79%
2017	0.021	0.027	0.026	0.128	0.044	0.026	0.017	27.90%	36.26%	35.83%
2018	0.026	0.029	0.034	0.124	0.051	0.073	0.019	29.65%	32.79%	37.56%

Note: **G*_w_***, **G*_nb_***, **G*_t_*** represent the intra-regional difference, the inter-regional difference and the hyper- variance density, respectively.

**Table 7:** Gini coefficients and contribution rates of the eight regions.

Year	Nationwide			Gini Coefficient by Region								Contribution Rate (%)		
	G*_w_*	G*_nb_*	G*_t_*	1	2	3	4	5	6	7	8	G*_w_*	G*_nb_*	G*_t_*
1997	0.011	0.057	0.039	0.052	0.019	0.112	0.010	0.143	0.147	0.091	0.114	10.27%	53.44%	36.29%
1998	0.010	0.052	0.038	0.050	0.022	0.103	0.009	0.138	0.139	0.070	0.120	10.16%	52.03%	37.81%
1999	0.010	0.050	0.035	0.052	0.022	0.025	0.012	0.152	0.135	0.075	0.116	10.22%	53.08%	36.70%
2000	0.009	0.053	0.030	0.058	0.012	0.024	0.009	0.136	0.124	0.089	0.103	10.26%	57.04%	32.70%
2001	0.009	0.053	0.026	0.071	0.004	0.025	0.011	0.133	0.088	0.074	0.126	10.07%	60.18%	29.75%
2002	0.007	0.046	0.021	0.068	0.005	0.032	0.007	0.123	0.016	0.079	0.102	9.92%	62.02%	28.06%
2003	0.009	0.050	0.029	0.069	0.005	0.124	0.012	0.118	0.015	0.100	0.091	9.84%	57.30%	32.86%
2004	0.007	0.056	0.016	0.067	0.001	0.111	0.017	0.136	0.010	0.055	0.064	8.80%	70.56%	20.64%
2005	0.008	0.060	0.022	0.066	0.008	0.101	0.025	0.171	0.016	0.079	0.058	9.12%	66.79%	24.08%
2006	0.007	0.064	0.014	0.069	0.010	0.103	0.025	0.178	0.015	0.042	0.036	8.33%	75.06%	16.62%
2007	0.007	0.059	0.014	0.067	0.011	0.096	0.023	0.181	0.017	0.047	0.012	8.40%	73.93%	17.67%
2008	0.007	0.060	0.016	0.070	0.011	0.099	0.024	0.167	0.019	0.049	0.030	8.60%	72.43%	18.97%
2009	0.008	0.064	0.017	0.068	0.011	0.098	0.021	0.162	0.082	0.042	0.036	8.71%	71.85%	19.45%
2010	0.010	0.084	0.024	0.081	0.063	0.100	0.019	0.174	0.132	0.038	0.098	8.75%	70.88%	20.37%
2011	0.006	0.059	0.012	0.088	0.010	0.086	0.014	0.157	0.023	0.037	0.013	8.23%	75.75%	16.02%
2012	0.010	0.080	0.023	0.096	0.072	0.092	0.009	0.158	0.156	0.032	0.065	8.85%	70.72%	20.43%
2013	0.010	0.084	0.019	0.093	0.059	0.090	0.008	0.140	0.183	0.032	0.071	8.69%	74.31%	17.01%
2014	0.008	0.066	0.018	0.096	0.044	0.098	0.015	0.142	0.085	0.029	0.038	8.73%	71.25%	20.02%
2015	0.009	0.076	0.017	0.092	0.013	0.105	0.012	0.138	0.180	0.023	0.034	8.51%	75.02%	16.47%
2016	0.008	0.072	0.017	0.080	0.014	0.112	0.012	0.131	0.173	0.020	0.040	8.52%	74.00%	17.48%
2017	0.006	0.052	0.015	0.073	0.017	0.115	0.017	0.126	0.030	0.019	0.047	8.48%	70.65%	20.87%
2018	0.008	0.061	0.020	0.073	0.019	0.108	0.022	0.121	0.145	0.020	0.050	9.08%	68.34%	22.58%

Note: 1, 2, 3, 4, 5, 6, 7, 8 represents the northern coast, northeast, eastern coast, middle Yellow River, southern coast, northwest, southwest, and middle Yangtze River.

**Table 8:** Parameters associated with GTWR.

Order	Parameter Name	Value
1	Neighbor	26
2	Residual Squares	1.3825
3	Sigma	0.0458
4	AICc	−2196.1
5	R^2^	0.6422
6	Adjusted R^2^	0.6389
7	Spatio-temporal Distance Ratio	0.1000

**Table 9:** GTWR results for 30 Chinese provinces in 1997 and 2018.

Region	UR		IS		ER		FDI		MI		WR		Intercept	
	1997	2018	1997	2018	1997	2018	1997	2018	1997	2018	1997	2018	1997	2018
Anhui	0.028	0.103	−0.200	−0.132	0.202	0.151	−0.459	−0.470	0.129	0.012	−0.094	−0.032	0.389	0.375
Beijing	−0.013	0.021	−0.049	−0.049	0.053	0.059	−0.084	−0.077	0.195	0.166	−0.153	−0.154	0.068	0.059
Fujian	0.121	0.130	−0.197	−0.166	0.014	−0.009	−0.281	−0.309	0.038	0.011	0.052	0.060	0.247	0.262
Gansu	0.335	0.320	0.089	0.162	0.064	−0.027	0.090	0.045	−0.238	−0.152	0.062	0.020	−0.155	−0.173
Guangdong	0.160	0.150	−0.395	−0.389	0.048	0.039	−0.066	−0.037	−0.040	−0.082	−0.115	−0.106	0.380	0.383
Guangxi	0.267	0.228	−0.228	−0.248	−0.089	−0.066	−0.119	−0.070	−0.196	−0.192	−0.444	−0.419	0.699	0.661
Guizhou	0.111	0.054	−0.155	−0.161	−0.161	−0.151	−0.177	−0.099	−0.111	−0.127	−0.215	−0.212	0.501	0.483
Hainan	0.295	0.274	−0.178	−0.195	−0.008	0.004	−0.072	−0.026	−0.254	−0.271	−0.572	−0.547	0.770	0.742
Hebei	0.036	0.053	−0.044	−0.051	−0.002	−0.010	−0.116	−0.108	0.098	0.081	−0.129	−0.132	0.140	0.146
Henan	−0.027	−0.019	−0.073	−0.073	−0.002	−0.016	−0.137	−0.093	0.102	0.039	−0.156	−0.159	0.208	0.223
Heilongjiang	−0.202	−0.196	−0.053	−0.067	−0.024	−0.009	0.184	0.225	−0.077	−0.126	0.018	−0.028	0.064	0.100
Hubei	−0.100	−0.065	−0.229	−0.157	0.229	0.150	−0.324	−0.248	0.255	0.168	−0.051	−0.015	0.238	0.172
Hunan	0.039	0.056	−0.252	−0.178	0.148	0.080	−0.249	−0.212	0.177	0.097	0.070	0.089	0.130	0.101
Jilin	−0.066	−0.071	−0.108	−0.118	0.035	0.041	0.125	0.148	−0.146	−0.179	−0.086	−0.115	0.171	0.203
Jiangsu	0.138	0.220	−0.111	−0.089	0.166	0.146	−0.556	−0.643	0.019	−0.047	−0.063	0.001	0.424	0.457
Jiangxi	0.080	0.121	−0.242	−0.144	0.144	0.071	−0.369	−0.397	0.187	0.100	0.092	0.135	0.177	0.161
Liaoning	−0.012	−0.012	−0.098	−0.113	0.038	0.053	0.068	0.086	−0.111	−0.130	−0.171	−0.185	0.208	0.221
Inner Mongolia	0.095	0.104	−0.038	−0.047	−0.062	−0.065	−0.003	0.004	−0.006	−0.014	−0.072	−0.071	0.089	0.091
Ningxia	0.188	0.180	0.064	0.077	0.001	−0.022	0.142	0.121	−0.211	−0.192	−0.013	−0.026	−0.052	−0.044
Qinghai	0.397	0.355	0.030	0.136	0.000	−0.129	0.057	0.015	−0.251	−0.177	0.042	−0.012	−0.088	−0.090
Shandong	−0.059	−0.059	−0.070	−0.084	0.022	0.016	−0.166	−0.123	0.149	0.084	−0.219	−0.234	0.236	0.272
Shanxi	0.082	0.092	−0.025	−0.032	−0.047	−0.055	−0.084	−0.083	0.046	0.042	−0.080	−0.084	0.110	0.116
Shaanxi	0.066	0.068	−0.060	−0.054	−0.028	−0.040	0.006	0.013	−0.050	−0.061	−0.032	−0.037	0.094	0.098
Shanghai	0.262	0.298	−0.015	−0.030	−0.007	0.019	−0.579	−0.757	−0.013	0.001	0.046	0.066	0.328	0.435
Sichuan	0.116	0.089	−0.059	−0.009	−0.009	−0.069	−0.064	−0.048	−0.103	−0.073	0.083	0.054	0.064	0.051
Tianjin	−0.030	0.013	−0.047	−0.050	0.042	0.056	−0.035	−0.054	0.174	0.131	−0.182	−0.176	0.067	0.082
Xinjiang	0.047	0.047	−0.104	−0.102	−0.203	−0.205	−0.042	−0.043	0.056	0.056	−0.109	−0.109	0.192	0.192
Yunnan	0.101	0.066	−0.261	−0.254	−0.135	−0.136	−0.076	−0.035	−0.095	−0.098	−0.159	−0.172	0.439	0.434
Zhejiang	0.277	0.311	−0.036	−0.026	−0.017	0.008	−0.542	−0.724	−0.023	0.004	0.079	0.094	0.295	0.379
Chongqing	0.109	0.051	−0.042	−0.027	−0.076	−0.096	−0.215	−0.141	−0.088	−0.075	0.019	−0.002	0.224	0.204

## Data Availability

The data presented in this study are available from the corresponding author.

## References

[1] Li Z., Li D., Yang W., Qi X. (2020). The spatial-temporal evolution and spatial convergence of ecological total factor productivity in China. Energy Environ..

[2] Wang K., Miao Z., Zhao M., Miao C., Wang Q. (2019). China’s provincial total-factor air pollution emission efficiency evaluation, dynamic evolution and influencing factors. Ecol. Indic..

[3] Yao L., Yu Z., Wu M., Ning J., Lv T. (2021). The Spatiotemporal Evolution and Trend Prediction of Ecological Wellbeing Performance in China. Land.

[4] Li J., Luo Y., Wang S. (2019). Spatial effects of economic performance on the carbon intensity of human well-being: The environmental Kuznets curve in Chinese provinces. J. Clean. Prod..

[5] Bei J. (2018). Study on the “high-quality development” economics. China Polit. Econ..

[6] Long X., Yu H., Sun M., Wang X., Klemeš J.J., Xie W., Wang C., Li W., Wang Y. (2020). Sustainability evaluation based on the three-dimensional ecological footprint and human development index: A case study on the four island regions in China. J. Environ. Manag..

[7] Daly H.E. (2005). Economics in a full world. Sci. Am..

[8] Victor P. (2010). Questioning economic growth. Nature.

[9] Daly H.E. (1974). The world dynamics of economic growth: The economics of the steady state. Am. Econ. Rev..

[10] Hou J., Ruan X., Lv J., Guo H. (2020). Two-Stage Super-Efficiency Slacks-Based Model to Assess China’s Ecological Wellbeing. Int. J. Environ. Res. Public Health.

[11] Daly H.E. (1996). Beyond Growth: The Economics of Sustainable Development.

[12] Moran D.D., Wackernagel M., Kitzes J.A., Goldfinger S.H., Boutaud A. (2008). Measuring sustainable development—Nation by nation. Ecol. Econ..

[13] Mandan Z., Dajian Z., Guoping L. (2013). Ecological well-being performance: Concept, connotation and empirical of G20. China Popul. Resour. Environ..

[14] Daly H.E. (1991). Towards an environmental macroeconomics. Land Econ..

[15] Kalimeris P., Bithas K., Richardson C., Nijkamp P. (2020). Hidden linkages between resources and economy: A “Beyond-GDP” approach using alternative welfare indicators. Ecol. Econ..

[16] Common M. (2007). Measuring national economic performance without using prices. Ecol. Econ..

[17] UNDP (1990). Human Development Report 1990: Concept and Measurement of Human Development.

[18] Hickel J. (2020). The sustainable development index: Measuring the ecological efficiency of human development in the anthropocene. Ecolog. Econ..

[19] Dietz T., Rosa E.A., York R. (2012). Environmentally efficient well-being: Is there a Kuznets curve?. Appl. Geogr..

[20] Cobb C.W., Daly H. (1989). The Index for Sustainable Economic Welfare.

[21] Malay O.E. (2021). How to articulate beyond GDP and businesses’ social and environmental indicators?. Soc. Indic. Res..

[22] Rees W.E. (1992). Ecological footprints and appropriated carrying capacity: What urban economics leaves out. Environ. Urban..

[23] Wackernagel M., Rees W. (1998). Our Ecological Footprint: Reducing Human Impact on the Earth.

[24] Haberl H., Erb K.-H., Krausmann F. (2001). How to calculate and interpret ecological footprints for long periods of time: The case of Austria 1926–1995. Ecol. Econ..

[25] McDonald G.W., Patterson M.G. (2004). Ecological footprints and interdependencies of New Zealand regions. Ecol. Econ..

[26] Yin Y., Han X., Wu S. (2017). Spatial and temporal variations in the ecological footprints in Northwest China from 2005 to 2014. Sustainability.

[27] Solarin S.A., Bello M.O. (2018). Persistence of policy shocks to an environmental degradation index: The case of ecological footprint in 128 developed and developing countries. Ecol. Indic..

[28] Feng Y., Zhong S., Li Q., Zhao X., Dong X. (2019). Ecological well-being performance growth in China (1994–2014): From perspectives of industrial structure green adjustment and green total factor productivity. J. Clean. Prod..

[29] Abdallah S., Thompson S., Michaelson J., Marks N., Steuer N. (2009). The Happy Planet Index 2.0: Why Good Lives Don’t Have to Cost the Earth.

[30] Zhang S., Zhu D., Shi Q., Cheng M. (2018). Which countries are more ecologically efficient in improving human well-being? An application of the Index of Ecological Well-being Performance. Resour. Conserv. Recycl..

[31] Dietz T., Rosa E.A., York R. (2009). Environmentally efficient well-being: Rethinking sustainability as the relationship between human well-being and environmental impacts. Hum. Ecol. Rev..

[32] Bian J., Ren H., Liu P. (2020). Evaluation of urban ecological well-being performance in China: A case study of 30 provincial capital cities. J. Clean. Prod..

[33] Ibrahim M.D., Alola A.A., Ferreira D.C. (2021). A two-stage data envelopment analysis of efficiency of social-ecological systems: Inference from the sub-Saharan African countries. Ecol. Indic..

[34] Wang R., Feng Y. (2020). Research on China’s Ecological Welfare Performance Evaluation and Improvement Path from the Perspective of High-Quality Development. Math. Probl. Eng..

[35] Zhang S., Zhu D., Zhang J., Li L. (2020). Which influencing factors could reduce ecological consumption? Evidence from 90 countries for the time period 1996–2015. Appl. Sci..

[36] Bian J., Zhang Y., Shuai C., Shen L., Ren H., Wang Y. (2020). Have cities effectively improved ecological well-being performance? Empirical analysis of 278 Chinese cities. J. Clean. Prod..

[37] Tone K. (2002). A slacks-based measure of super-efficiency in data envelopment analysis. Eur. J. Oper. Res..

[38] Dagum C. (1997). A new approach to the decomposition of the Gini income inequality ratio. Empir. Econ..

[39] Tian P., Li J., Wang L., Liu R., Shi X. (2020). Dynamics of three-dimensional ecological footprint of Zhejiang coastal zone and its influencing factors based on GTWR model. J. Appl. Ecol..

[40] Wu B., Li R., Huang B. (2014). A geographically and temporally weighted autoregressive model with application to housing prices. Int. J. Geogr. Inf. Sci..

[41] Chu H., Huang B., Lin C.-Y. (2015). Modeling the spatio-temporal heterogeneity in the PM10-PM2. 5 relationship. Atmos. Environ..

[42] Liu T., Li J., Chen J., Yang S. (2019). Urban ecological efficiency and its influencing factors—A case study in Henan province, China. Sustainability.

[43] Dociu M., Dunarintu A. (2012). The socio-economic impact of urbanization. Int. J. Acad. Res. Account. Financ. Manag. Sci..

[44] Wang Z., Liang L., Sun Z., Wang X. (2019). Spatiotemporal differentiation and the factors influencing urbanization and ecological environment synergistic effects within the Beijing-Tianjin-Hebei urban agglomeration. J. Environ. Manag..

[45] Liu X., Bae J. (2018). Urbanization and industrialization impact of CO_2_ emissions in China. J. Clean. Prod..

[46] Behera S.R., Dash D.P. (2017). The effect of urbanization, energy consumption, and foreign direct investment on the carbon dioxide emission in the SSEA (South and Southeast Asian) region. Renew. Sustain. Energy Rev..

[47] Tang C.F., Tan B.W. (2015). The impact of energy consumption, income and foreign direct investment on carbon dioxide emissions in Vietnam. Energy.

[48] Reinhard S., Lovell C.K., Thijssen G. (2000). Environmental efficiency with multiple environmentally detrimental variables; estimated with SFA and DEA. Eur. J. Oper. Res..

[49] Ma L., Long H., Chen K., Tu S., Zhang Y., Liao L. (2019). Green growth efficiency of Chinese cities and its spatio-temporal pattern. Resour. Conserv. Recycl..

[50] Wang S., Zhang Y., Wen H. (2021). Comprehensive Measurement and Regional Imbalance of China’s Green Development Performance. Sustainability.

[51] Song Y., Mei D. (2021). Sustainable development of China’s regions from the perspective of ecological welfare performance: Analysis based on GM (1,1) and the malmquist index. Environ. Dev. Sustain..

[52] Wang R. (2020). The influence of environmental regulation on the efficiency of China’s regional green economy based on the gMM model. Pol. J. Environ. Stud..

[53] Fang C., Yu D. (2017). Urban agglomeration: An evolving concept of an emerging phenomenon. Landsc. Urban Plan..

[54] Yin S., Dongjie G., Weici S., Weijun G. (2017). Integrated assessment and scenarios simulation of urban water security system in the southwest of China with system dynamics analysis. Water Sci. Technol..

[55] Costanza R., Hart M., Talberth J., Posner S. (2009). Beyond GDP: The Need for New Measures of Progress.

[56] Stiglitz J.E., Sen A., Fitoussi J. (2010). Mismeasuring Our Lives: Why GDP Doesn’t Add up.

[57] Kubiszewski I., Costanza R., Franco C., Lawn P., Talberth J., Jackson T., Aylmer C. (2013). Beyond GDP: Measuring and achieving global genuine progress. Ecolog. Econ..

[58] Tang J.B. (2020). Three Issues of Ecological Welfare—Based on the Perspective of Ecological Values. J. Nanchang Univ. (Hum. Soc. Sci.).

[59] Doloreux D., Parto S. (2005). Regional innovation systems: Current discourse and unresolved issues. Technol. Soc..

[60] Zhong Y., Wu S. (2021). Spatio-temporal Pattern Evolution and Impact Factors of People’s Livelihood Developing Undertakings Level in China. J. Nanchang Univ. (Hum. Soc. Sci.).

